# YRNAs: New Insights and Potential Novel Approach in Head and Neck Squamous Cell Carcinoma

**DOI:** 10.3390/cells9051281

**Published:** 2020-05-21

**Authors:** Kacper Guglas, Tomasz Kolenda, Maciej Stasiak, Magda Kopczyńska, Anna Teresiak, Matthew Ibbs, Renata Bliźniak, Katarzyna Lamperska

**Affiliations:** 1 Laboratory of Cancer Genetics, Greater Poland Cancer Centre, Poznan, Poland, ul. Garbary 15, 61-866 Poznan, Poland; kolenda.tomek@gmail.com (T.K.); mg.kopczynska@gmail.com (M.K.); anna.teresiak@wco.pl (A.T.); renata.blizniak@wco.pl (R.B.); 2 Postgraduate School of Molecular Medicine, Medical University of Warsaw, ul. Zwirki 61 and ul. Wigury, 02-091 Warsaw, Poland; 3 Department of Cancer Immunology, Chair of Medical Biotechnology, Poznan University of Medical Sciences, ul. Rokietnicka 8, 60-101 Poznan, Poland; 4 Chair of Medical Biotechnology, Poznan University of Medical Sciences, ul. Rokietnicka 8, 60-101 Poznan, Poland; maciej.stasiak96@gmail.com; 5 Department of Tumour Pathology, Greater Poland Cancer Centre, Poznan, Poland, ul. Garbary 15, 61-866 Poznan, Poland; matthev.ibbs@wco.pl; 6 Chair and Department of Tumour Pathology and Prophylaxis, Poznan University of Medical Sciences, ul. Garbary 15, 61-866 Poznan, Poland

**Keywords:** YRNA, RNY, small non-coding RNA, Ro60 associated YRNA, HNSCC, biomarker, GSEA, cancer

## Abstract

YRNAs are a class of non-coding RNAs that are components of the Ro60 ribonucleoprotein particle and are essential for initiation of DNA replication. Ro60 ribonucleoprotein particle is a target of autoimmune antibodies in patients suffering from systemic lupus erythematosus and Sjögren’s syndrome. Deregulation of YRNAs has been confirmed in many cancer types, but not in head and neck squamous cell carcinoma (HNSCC). The main aim of this study was to determine the biological role of YRNAs in HNSCC, the expression of YRNAs, and their usefulness as potential HNSCC biomarkers. Using quantitative reverse transcriptase (qRT)-PCR, the expression of YRNAs was measured in HNSCC cell lines, 20 matched cancer tissues, and 70 FFPETs (Formaline-Fixed Paraffin-Embedded Tissue) from HNSCC patients. Using TCGA (The Cancer Genome Atlas) data, an analysis of the expression levels of selected genes, and clinical-pathological parameters was performed. The expression of low and high YRNA1 expressed groups were analysed using gene set enrichment analysis (GSEA). YRNA1 and YRNA5 are significantly downregulated in HNSCC cell lines. YRNA1 was found to be significantly downregulated in patients’ tumour sample. YRNAs were significantly upregulated in T4 stage. YRNA1 showed the highest sensitivity, allowing to distinguish healthy from cancer tissue. An analysis of TCGA data revealed that expression of YRNA1 was significantly altered in the human papilloma virus (HPV) infection status. Patients with medium or high expression of YRNA1 showed better survival outcomes. It was noted that genes correlated with YRNA1 were associated with various processes occurring during cancerogenesis. The GSEA analysis showed high expression enrichment in eight vital processes for cancer development. YRNA1 influence patients’ survival and could be used as an HNSCC biomarker. YRNA1 seems to be a good potential biomarker for HNSCC, however, more studies must be performed and these observations should be verified using an in vitro model.

## 1. Introduction

Head and neck squamous cell carcinoma (HNSCC) is the sixth most common cancer worldwide. It occurs in the epithelial tissues of the aerodigestive tract [1]. Tumours may be divided according to their localization: tongue, oral, laryngeal, or nasopharyngeal squamous cell carcinomas (TSCC, OSCC, LSCC, and NSCC, respectively) [2]. Tobacco smoking, alcohol consumption, and human papilloma virus (HPV) infections are the most common risk factors. HPV infection is a very important risk factor among younger patients and correlates with better tumour prognosis and treatment outcomes [3]. HNSCC development involves genetic factors such as single mutations or chromosomal aberrations, as well as epigenetic factors such as expression changes among regulatory RNAs, which are responsible for many important processes for example, cell cycle regulation, apoptosis, proliferation, and cell migration. Changes in these genes result in the loss of their functions and the development of disease [4]. Commonly used therapies involve surgical resection, radiotherapy, chemotherapy, or a combination of these [1,2,3,4]. Unfortunately, owing to high resistance to radio and chemotherapy, as well as a high capacity for both local and remote metastasis, standard treatments are often ineffective, leading to high mortality rates among HNSCC patients [2]. It is crucial to find new approaches in personalized medicine for the treatment of HNSCC, and knowledge of molecular biology may be of help in developing these new therapies.

Analysis of protein-coding genes (sequence changes) and their transcripts (alternative splicing forms, RNA editing) has not led to spectacular improvements in HNSCC treatment and outcome [1,2,3]. Because of this lack of progress, the search for new treatment strategies and biomarkers in oncology now focusses on the analysis and usage of non-coding RNAs (ncRNAs) [1,2,3]. Most of the human genome, about 98%, consists of ncRNAs [1,2,3,4,5,6,7], and they have important roles in many cellular processes [1,2,3,4,5,6,7]. ncRNAs can be divided into two groups: small ncRNAs (smaller than 200 nucleotides, for example, miRNAs) and long ncRNAs (longer than 200 nucleotides, for example, lncRNAs) [5,6,7]. Neither of these groups code proteins, though they play crucial roles in the regulation of protein coding RNAs as well as other ncRNAs [5,6]. One group of small ncRNAs are YRNAs (Ro-associated Y), consisting of approximately 80–110 nucleotides, and they are components of the Ro60 ribonucleoprotein particle [5,6,7,8]. Among YRNAs, we can distinguish between four highly conserved types of YRNA transcripts: YRNA1 (RNY1), YRNA3 (RNY3), YRNA4 (RNY4), and YRNA5 (RNY5). In humans, these four YRNA genes form a cluster at a single chromosomal locus on chromosome 7q36 [8]. RNA polymerase III transcribes these genes [6], the products of which comprise of a stem-loop structure, an internal loop, and a polyuridine tail [5,6,7,8]. The nucleotide sequences of the upper and lower domains are highly conserved, though the nucleotide sequence of the internal loop varies greatly between individual YRNAs. Loop domains are the least conserved elements and modulate chromatin association [8]. The upper stem is essential for the initiation of DNA replication. The lower stem is an Ro60 binding site, forming an activated protein–ribonucleoprotein complex, which is a target of the immune system in patients suffering from autoimmune diseases such as systemic lupus erythematosus or Sjögren’s syndrome [8]. The lower stem also controls nuclear export of YRNAs [8]. The polyuridine tail, found at the end of YRNAs, is an LA protein-binding site, which is an autoantigen found to be complexed with a subset of Ro60 ribonucleoproteins. LA protein is essential for the efficient termination of RNA polymerase III transcription [8].

Few reports have indicated a connection between YRNAs and cancer. Deregulation of YRNAs is crucial for the carcinogenesis of prostate cancer [5], and their overexpression has been reported in breast cancer [6], head and neck squamous cell carcinoma [9], and clear cell renal cell carcinoma, where inhibition of YRNA1 and YRNA3 was shown to decrease cell proliferation [6]. In bladder cancer, expression of YRNAs predicts survival of patients [7].

Interestingly, YRNAs detected in the serum and plasma have made them a potential circulating biomarker in various diseases [5,6,7].

Recent studies also show that YRNAs are processed in apoptotic and lipid-laden macrophages into small sequences called YRNA-derived small RNAs [10,11]. These sequences are approximately 24 to 34 nucleotides in length and are mapped to the end of the stem regions [10,11]. YRNA-derived small RNAs are processed into microRNA-like small RNAs, however, their function has not yet been examined [9]. As YRNA-derived small RNAs are highly abundant in cells, tissues, and body fluids, they are of interest to scientists as potential disease biomarkers [8].

In this study, the biological role of YRNAs in HNSCC was evaluated, as was their usefulness as biomarkers or putative therapeutic targets.

## 2. Materials and Methods

### 2.1. HNSCC Cell Lines

The HNSCC cell lines SCC-040 (oral cancer model), SCC-25 (tongue cancer model), FaDu (hypopharyngeal cancer model), Cal27 (tongue cancer model), and DOK (dysplastic oral keratinocyte cells from a tongue as a model of healthy tissue) [12] were used for the study. The SCC-040 and SCC-25 cell lines were maintained according to the instructions from the DSMZ (Deutsche Sammlung von Mikroorganismen und Zellkulturen GmbH, Leibniz Institut, Germany). The FaDu cell line was cultured as described previously [13]. The Cal27 cell line was maintained according to the instructions from the ATCC (American Type Culture Collection, USA) and the DOK cell line, as described by Sigma-Aldrich. All cell lines were cultured with penicillin-streptomycin antibiotics (Merck Millipore) and mycoplasma detection tests were performed routinely using the VenorGeM Mycoplasma PCR Detection Kit (Minerva Biolabs).

### 2.2. Patient Samples

Tested tissues originated from HNSCC patients, who were surgically treated in the Greater Poland Cancer Centre in 2010 and 2011. The studied material included 20 matched cancer tissues and normal epithelial tissues collected at least 2 cm from the tumour’s margins. As a validation cohort, 70 FFPETs of HNSCC patients were collected.

None of the patients had received preoperative chemotherapy or radiotherapy, nor had they been diagnosed with local recurrences or second primary tumours prior to sample collection. All tumours were histologically confirmed as squamous cell carcinomas and as HPV negative. The tumour differentiation grade was determined according to the World Health Organisation (WHO) criteria and the TNM classification was in accordance with recommendations of the Union for International Cancer Control (UICC).

### 2.3. RNA Isolation and Quantitative RT-PCR

Total RNA from the cell lines was isolated using a High Pure miRNA isolation kit (Roche), according to the isolation protocol for total RNA from cell line samples.

For the discovery cohort study, HNSCC tissues were frozen at or below −80 °C immediately after surgery and total RNA was isolated using TRI reagent (Sigma Aldrich). Next, samples were concentrated and purified using the High Pure miRNA Isolation Kit (Roche) according to the protocol for isolation of total RNA. For the validation cohort study, FFPETs with HNSCC were isolated using the High Pure FFPET Isolation Kit (Roche) according to the protocol for isolation of total RNA.

RNA was quantified using a NanoDrop 2000 spectrophotometer (ThermoScientific) and stored at −80 °C until use.

Complementary DNA was synthesized using an iScript cDNA Synthesis Kit (Bio-Rad) and 0.5 μg of the total RNA was used. Quantitative PCR was performed using 2x concentrated SYBR Green Master Mix (Roche) with specific primers to detect YRNA1, YRNA3, YRNA4, and YRNA5, as described previously [5,6,7]. Endogenous controls HPRT1 (F: 5′-TGA CCT TGA TTT ATT TTG CAT ACC-3′ AND R: 5′-CGA GCA AGA CGT TCA GTC CT-3′) and B2M (F: 5′-TTC TGG CCT GGA GGC TAT C-3′ and R: 5′-TCA GGA AAT TTG ACT TTC CAT TC-3′) were used at a final reaction concentration of 0.5 μM with 5x diluted cDNA. The real-time PCR reactions were performed on a LightCycler 96 (Roche) device, and a melting curve was performed to discriminate between non-specific products of the PCR reaction. All real-time PCR data were analysed by calculating the 2-ΔC_T_, normalizing against the mean of HPRT1 and B2M expression.

### 2.4. TCGA Data

Clinical data of HNSCC patients and expression values of selected genes were obtained from cBioPortal (Head and Neck Squamous Cell Carcinoma, TCGA, Provisional, 530 samples). The expression data of miRNAs for HNSCC patients were downloaded from the Santa Cruz University of California data set https://xenabrowser.net/datapages/?dataset=TCGA-HNSC.mirna.tsv&host=https%3A%2F%2Fgdc.xenahubs.net&removeHub=https%3A%2F%2Fxena.treehouse.gi.ucsc.edu%3A443 (stem loop expression-miRNA expression quantification; RNAseq log2(RPM + 1), 569 samples). All data are available online and access is unrestricted.

### 2.5. Interaction between YRNA1 Expression and Other Genes

The correlation of YRNA1 expression with other genes was examined using StarBase v3.0 (http://starbase.sysu.edu.cn/) and the heat maps were made using MORPHEUS Broad Institute (https://software.broadinstitute.org/morpheus/). Statistical analysis was performed using GraphPad Prism 5 software (GraphPad Software San Diego, CA, USA). For the correlation, *p*-value < 0.05 was considered as significant. Tumor metastasis, cell cycle, cancer stem cells, apoptosis, and epithelial to mesenchymal transition (EMT) were chosen as the most interesting processes that conclude to developing a tumour.

### 2.6. Functional Enrichment Analysis and Prediction of Gene Function

Gene set enrichment analysis (GSEA) software version 3.0 (http://www.gsea-msigdb.org/gsea/index.jsp) was used as previously described for analysis of functional enrichment [14,15]. HNSCC patients were divided into two groups with high and low expression of YRNA1. The input file contained expression data for 20530 genes and 117 patients. We used 1000 gene set permutations for the analysis and pathways (the oncogenic Signatures (C), hallmark gene set (H), and gene ontology (GO)) with a nominal *p*-value *p* ≤ 0.05 and FDR *q*-value ≤ 0.25 were considered significant. Next, the interactions between protein coding genes in the pathway which were the most significantly enriched in group of patients with low versus high expression of YRNA1 were analysed using the GeneMANIA prediction tool (http://genemania.org) [16].

### 2.7. Statistical Analysis

The clinical-pathological parameters analysed for associations between YRNA1, YRNA3, YRNA4, and YRNA5 expression levels at all localizations in the HNSCC samples included the following: age (below or above 62 years), sex (female vs. male), cancer stage (I + II vs. III + IV), T stage (T1 vs. T2 vs. T3 vs. T4), N stage (N0 + N1 vs. N2 + N3), grade (G1 + G2 vs. G3 + G4), perineural invasion (positive vs. negative), lymph node dissection (positive vs. negative), angiolymphatic invasion (positive vs. negative), disease surgical margin status (positive vs. negative), and HPV p16 status (positive vs. negative). The normality of the groups was tested using the Shapiro–Wilk test and, subsequently, comparison of the groups was carried out using the t-test or Mann–Whitney U test. Analysis of YRNA expression relative to tumour localizations (oral cavity, pharynx, and larynx) and T-stages was performed using the Shapiro–Wilk normality test and one-way analysis of variance (ANOVA), Kruskal–Wallis test, and post-test: additionally, Dunn’s multiple comparison test was used.

To discriminate between YRNA expression from cancer and normal samples, receiver operating characteristic (ROC) analysis was used and the area under the curve (AUC) was calculated.

Disease-free survival (DFS) and overall survival (OS) analyses were performed using two subgroups (low and medium + high), generated using 25% gene expression as the cut-off. Next, the subgroups were compared using log-rank (Mantel–Cox), Gehan–Breslow–Wilcoxon, and hazard ratio (Mantel–Haenszel; HR) tests. The 95% confidence interval (CI) of the ratio was calculated. In all analyses, *p* < 0.05 was used to determine statistical significance.

## 3. Results

### 3.1. Expression of YRNAs Is Changed in HNSCC Cell Lines and in Patients’ Tumours

The expression levels of YRNA1, YRNA3, YRNA4, and YRNA5 were measured in four different HNSCC cell lines, Cal27, FaDu, SCC-25, and SCC-040, and compared with the DOK cell line. The analysed cell lines are characterized by different tumorigenic potentials, among which FaDu is the most aggressive [11], and the DOK cell line was assumed to be a model of dysplastic oral mucosa cells of a partially transformed and non-malignant phenotype [10].

**Figure 1:**
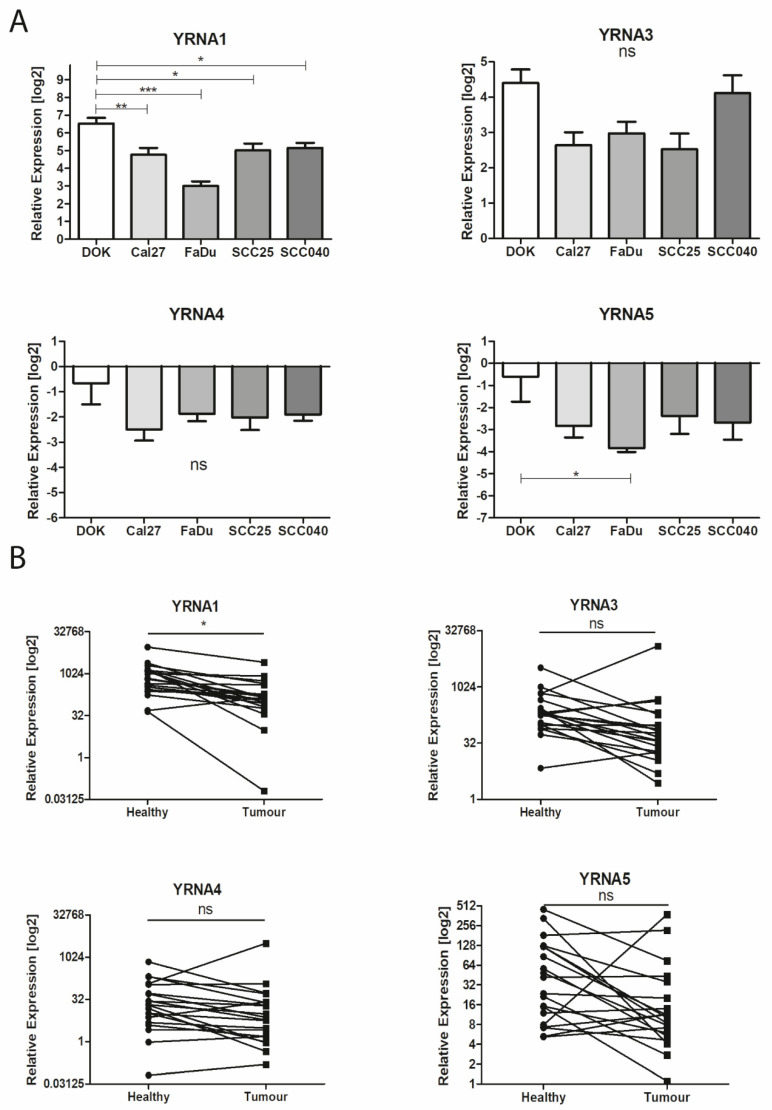
Expression levels of YRNA1, YRNA3, YRNA4, and YRNA5 in head and neck squamous cell carcinoma (HNSCC). (**A**) Dysplastic oral keratinocyte (DOK), Cal27 and SCC-040 cell lines, one-way analysis of variance (ANOVA) with Tukey’s multiple comparison post-test; the graphs show relative expression and means of value with SEM; (**B**) patients’ tumours and healthy tissue samples, paired T-test; * *p* < 0.05; ** *p* < 0.01; *** *p* < 0.001; ns—not significant.

Downregulation of YRNA1 in malignant cell lines, Figure 1A, of Cal27 (27.13 ± 8.352), FaDu (7.990 ± 1.561), SCC-25 (32.27 ± 9.728), and SCC-040 (35.50 ± 7.901) was observed in comparison with the DOK (92.15 ± 22.58) cell line (*p* = 0.0001). No significant differences were noticed between the expression of YRNA3 and YRNA4 in malignant cell lines and in DOK (*p* = 0.0797 and *p* = 0.1159 respectively). In the case of YRNA5, significant downregulation was observed only between DOK and FaDu (*p* = 0.0470), Figure 1A.

The expression levels of YRNA1, YRNA3, YRNA4, and YRNA5 were tested in 20 HNSCC patients’ tumours and in matched adjacent healthy tissues, Figure 1B. Only YRNA1 was found to be significantly downregulated in tumour samples compared with matched adjacent healthy tissues (1247 ± 440.9 vs. 322.8 ± 130.5; *p* = 0.0109). The expression levels for YRNA3 (420.8 ± 164.6 vs. 731.6 ± 631.7; *p* = 0.6317), YRNA4 (79.54 ± 35.41 vs. 176.6 ± 158.2; *p* = 0.5502), and YRNA5 (84.07 ± 26.40 vs. 43.78 ± 158.2; *p* = 0.2279) showed no significant differences between paired samples, Figure 1B.

**Figure 2:**
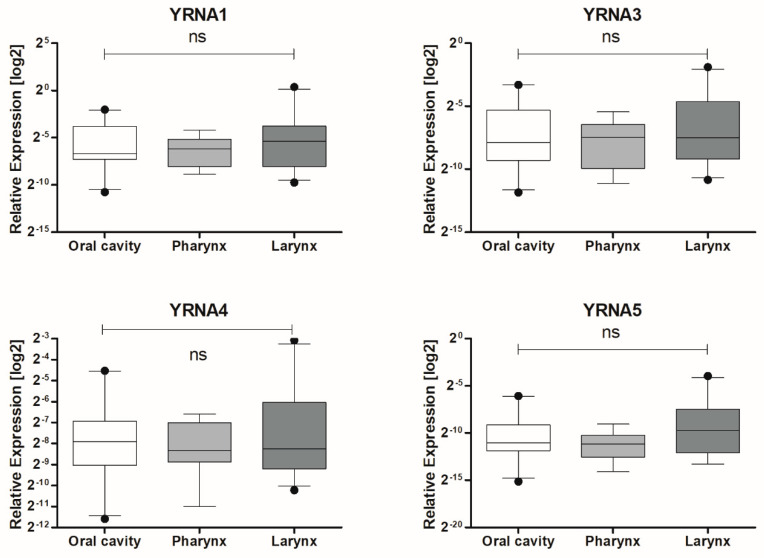
Divided according to the Expression levels of YRNA1, YRNA3, YRNA4, and YRNA5 in patients’ FFPET samples. Three main localizations of HNSCC tumours: oral cavity, pharynx, and larynx; one-way ANOVA with Kruskal–Wallis post-test or Dunn’s multiple comparison post-test; ns—not significant.

The expression levels of YRNAs were examined in cancer samples from 70 patients and compared according to the three main localization groups of HNSCC according to the National Institute of Health, Figure 2. No significant differences between oral cavity, pharynx, and larynx expression levels YRNA1 (0.04271 ± 0.01368 vs. 0.01781 ± 0.004761 vs. 0.1049 ± 0.05659; *p* = 0.4274), YRNA3 (0.02149 ± 0.007280 vs. 0.007132 ± 0.002129 vs. 0.03075 ± 0.01271; *p* = 0.5815), YRNA4 (0.009035 ± 0.002777 vs. 0.004548 ± 0.0009427 vs. 0.01377 ± 0.005407; *p* = 0.8417), and YRNA5 (0.002184 ± 0.0005904 vs. 0.0005768 ± 0.0001435 vs. 0.006071 ± 0.003009; *p* = 0.2573) were observed, Figure 2. Patient characteristics are presented in Table 1.

**Figure 3:**
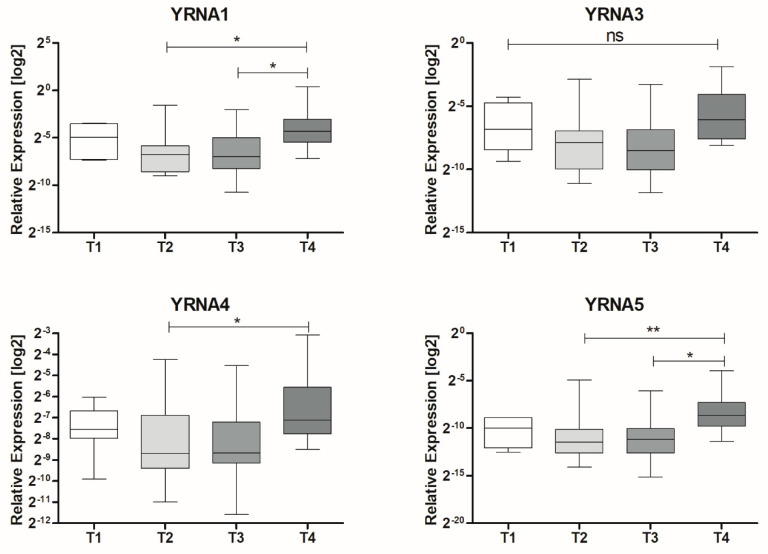
Expression levels of YRNA1, YRNA3, YRNA4, and YRNA5 in patients’ FFPET samples. Divided according to HNSCC T stage; one-way ANOVA with Kruskal–Wallis post-test or Dunn’s multiple comparison test; * *p* < 0.05; ** *p* < 0.01; ns—not significant.

The expression of YRNA1, YRNA3, YRNA4, and YRNA5 was also analysed according to patients’ T stage, Figure 3. T stage describes the primary tumour size and whether it has invaded tissues in the close range to the tumour. YRNA1 (*p* = 0.0211), YRNA3 (*p* = 0.0339), YRNA4 (*p* = 0.0357), and YRNA5 (*p* = 0.0071) were found to be significantly upregulated in stage T4 of HNSCC. Interestingly, YRNAs studied in HNSCC showed similar expression levels in stage T1 as in stage T4, Figure 3.

**Figure 4:**
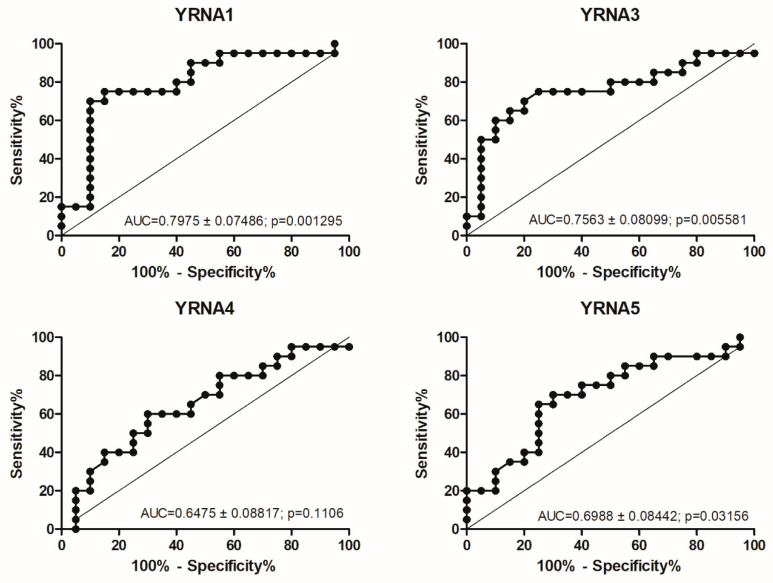
Receiver operating characteristic (ROC) analysis of YRNA1, YRNA3, YRNA4 and YRNA5. Examined in 20 paired patients’ samples; *p* < 0.05 considered to be significant. AUC, area under the curve.

ROC analysis was performed on patients’ tissues samples, paired healthy, and tumour samples, to specify the diagnostic potential of the analysed YRNAs, Figure 4. YRNA1 (AUC = 0.7975 ± 0.07486; *p* = 0.001295), YRNA3 (AUC = 0.7563 ± 0.08099; *p* = 0.005581), YRNA4 (AUC = 0.6475 ± 0.08817; *p* = 0.1106), and YRNA5 (AUC = 0.6988 ± 0.08442; *p* = 0.03156) showed similar values, however, the expression of YRNA1 showed the highest sensitivity result by percentage, making it the most specific of all examined YRNAs. YRNA4 showed the lowest sensitivity result by percentage, Figure 4.

### 3.2. TCGA Analysis Indicates That YRNA1 Levels Differ According to HPV Status

**Figure 5:**
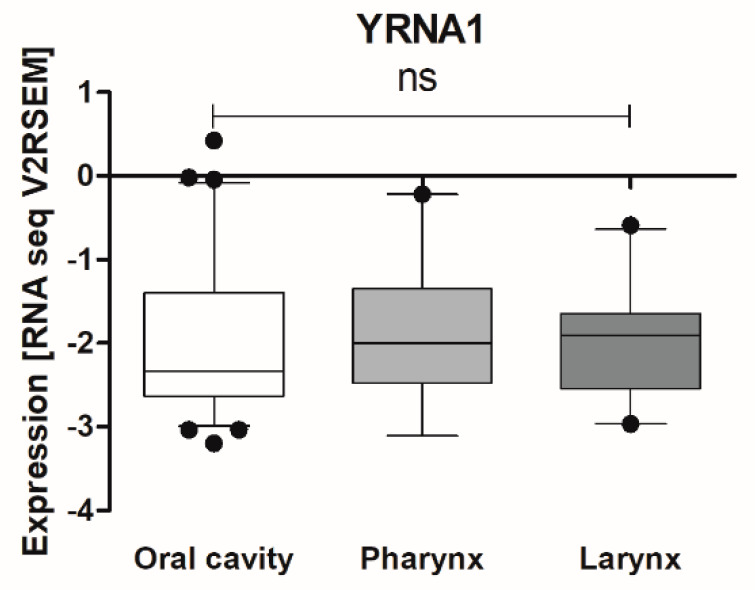
The expression levels of YRNA1 in three locations of HNSCC. Oral cavity (*n* = 76), pharynx (*n* = 19), and larynx (*n* = 21); one-way ANOVA with Dunn’s multiple comparisons post-test; ns—not significant.

Next, owing to the small number of patients (*n* = 20) and insufficient data, the expression levels of YRNA1 according to clinical-pathological parameters for 116 patients were analysed using available TCGA data, Figure 5. First, expression levels of YRNA1 were compared between the oral cavity (*n* = 76), pharynx (*n* = 19), and larynx (*n* = 21). Analysis showed no significant differences between the analysed localizations (−1.981 ± 0.1001 vs. −1.885 ± 0.1696 vs. −1.963 ± 0.1427; *p* = 0.5669), Figure 5. The remaining parameters, age (*p* = 0.7491), sex (*p* = 0.0932), alcohol consumption (*p* = 0.3472), tobacco smoking (*p* = 0.7405), cancer stage (*p* = 0.8773), T stage (*p* = 0.9603), N stage (*p* = 0.1597), grade (*p* = 0.9464), perineural invasion (*p* = 0.3927), lymph node dissection (*p* = 0.2735), angiolymphatic invasion (*p* = 0.3307), and disease surgical margin status (*p* = 0.3690), showed no statistical differences. Significantly higher expression of YRNA1 was noticed only when comparing HPV+ TCGA patients with HPV- patients (*p* = 0.0002), Table 2.

### 3.3. Higher Expression of YRNA1 Is Associated with Longer DFS and OS

**Figure 6:**
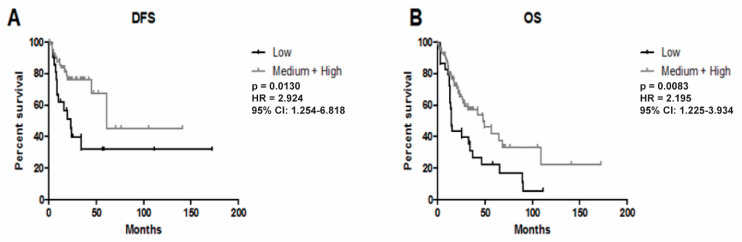
Influence of YRNA1 expression on HNSCC patients’ outcome. (**A**) Disease-free survival (DFS) and (**B**) overall survival (OS); *p* < 0.05 was considered to be significant. CI, confidence interval; HR, hazard ratio.

The HNSCC samples (*n* = 83 for DFS and *n* = 116 for OS) were divided into two groups based on the 25% percentile YRNA1 expression value, Figure 6. The low expression group for DFS analysis was defined as expression levels below −2.526 (*n* = 21) and the medium + high expression group included all samples above −2.526 (*n* = 62). It was found that patients with medium + high YRNA1 expression had longer DFS compared with the group with lower expression (*p* = 0.0130; HR = 2.924; 95% confidence interval (CI): 1.254–6.818; Figure 6A). The low expression group for OS analysis was defined as expression levels below −2.598 (*n* = 29) and the medium + high expression group included all samples above −2.598 (*n* = 87). The OS analysis showed that patients with medium + high YRNA1 expression had longer survival than the lower-expression group (*p* = 0.0083; HR = 2.195; 95% CI: 1.225–3.934; Figure 6B).

### 3.4. YRNA1 Expression Is Associated with Changes in the Expression of Many Important Genes

**Figure 7:**
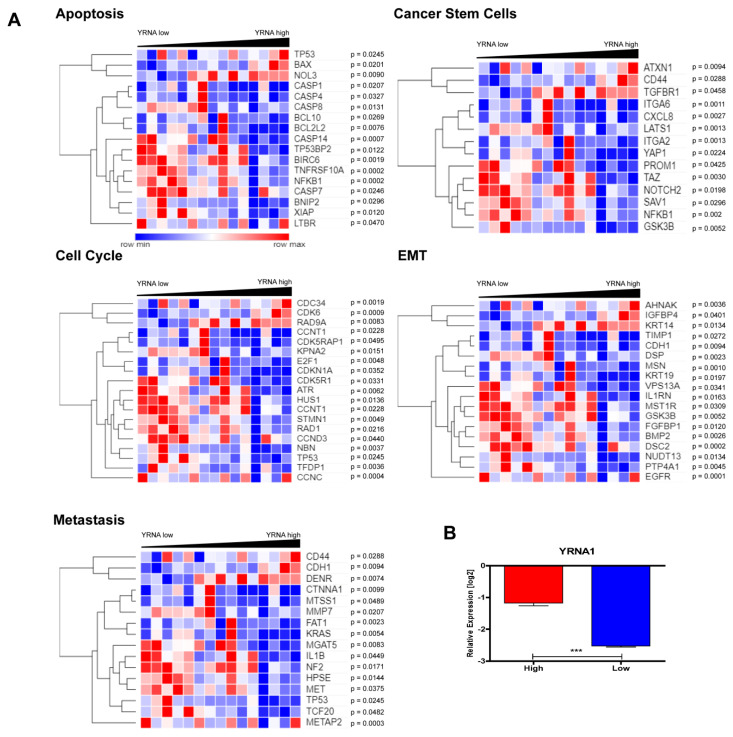
The expression of correlated genes with YRNA1 connected with different processes. (**A**) The expression of correlated genes with YRNA1 connected with tumour metastasis, cancer stem cell development, apoptosis, epithelial to mesenchymal transition (EMT), and the cell cycle in the group of HNSCC TCGA patients with high and low expression of YRNA1; T-test; Mann–Whitney test; *p* < 0.05 considered as significant. (**B**) A comparative analysis of patients with high YRNA1 expression and low by T-test; *p* < 0.05 considered as significant.

An analysis of the expression of correlated genes with YRNA1 expression was performed for crucial processes that occur in cancer cells using StarBase v3.0 and GraphPad Prism 5 software. The expression values of YRNA1 were derived from TCGA data and divided into low (*n* = 69) and high (*n* = 48) expression groups based on expression of −1.97016 of YRNA1 as the cut-off. For differences between groups, *p* < 0.05 was considered to be statistically significant, Figure 7A. It was noted that YRNA1 expression is potentially associated with tumour metastasis (16 changed genes out of 95 examined), cancer stem cell development (14 changed genes out of 88 examined), apoptosis (17 changed genes out of 172 examined), epithelial to mesenchymal transition (18 changed genes out of 157 examined), and cell cycle (18 changed genes out of 119 examined), Figure 7A. Furthermore, an analysis of patients with high expression of YRNA1 compared with patients with low expression of YRNA1 showed statistically significant differences between these two groups (*p* value < 0.0001), Figure 7B.

### 3.5. Patients with High and Low Expression of YRNA1 Have a Different Pattern of Genes

**Figure 8:**
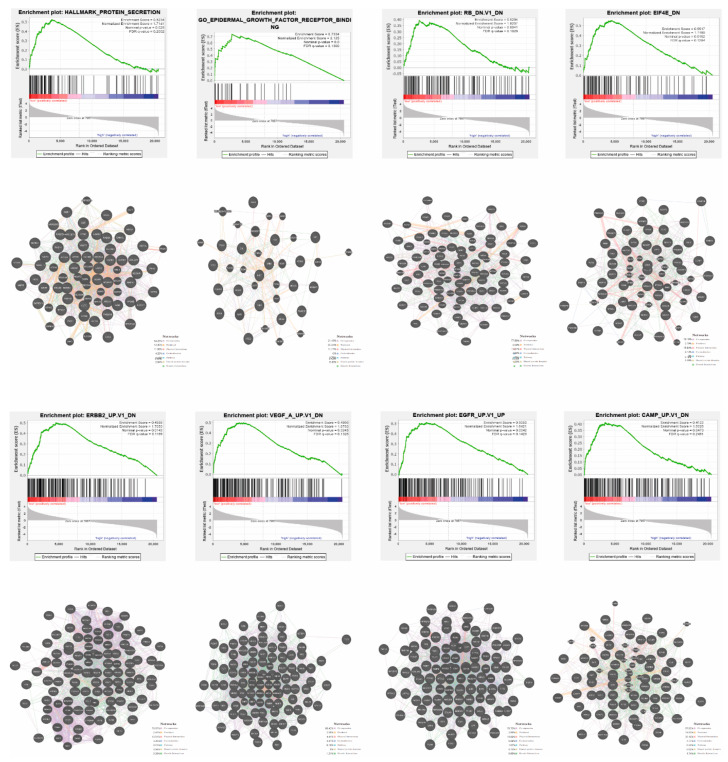
Gene set enrichment analysis (GSEA) and GeneMania analysis.

Functional implications of YRNA1 expression signature were analysed using gene set enrichment analysis (GSEA) and the eight top enriched datasets are shown in Figure 8. It was found that most upregulated genes in the YRNA1 low expressing group of patients are clustered most significantly in protein secretion, epidermal growth factor receptor binding, RB (the RB-dependent pathway), EIF4E (the EIF4E-dependent pathway), ERBB2 (the ERBB2-dependent pathway), VEGF (the VEGF-dependent pathway), EGFR (the EGFR-dependent pathway), and cAMP (the cAMP-dependent pathway) (normalized enrichment score (NES) = 1.7141, NES = 2.125, NES = 1.8207, NES = 1.7160, NES = 1.7050, NES = 1.6783, NES = 1.6421, and NES = 1.5026, respectively). Next, the interactions between protein coding genes in the pathways that were the most significantly enriched in the group of patients with low expression of YRNA1 were analysed using the GeneMANIA prediction tool. The following numbers of genes were indicated: protein secretion—53 genes, epidermal growth factor receptor binding—18 genes, RB-dependent pathway—71 genes, EIF4E-dependent pathway—51 genes, ERBB2-dependent pathway—83 genes, VEGF-dependent pathway—85 genes, EGFR-dependent pathway—81 genes, and cAMP-dependent pathway—64 genes, in which 64.23%, 21.19%, 77.56%, 70.16%, 78.87%, 88.42%, 78.70%, and 57.52% of them are co-expressed, respectively, Figure 8.

GeneMANIA plots and GSEA results of HNSCC patients analyzed in groups with low (red)/high (blue) expression of YRNA1 and interactions between protein-encoding genes in the pathways, which were the most enriched in a group of patients with low YRNA1. GSEA plots of the most enriched datasets with p-value <0.05 (nominal p-value), FDR q-value <0.25 (false discovery rate) and with NES (normalized enrichment score) were shown.

## 4. Discussion

Head and neck squamous cell carcinomas (HNSCCs) are a group of cancers associated with many difficulties in successful treatment. One of the main reasons for such difficulties in the application of standard therapies such as chemotherapy, radiotherapy, or their combination is the high resistance of HNSCC cells to radio and chemotherapy [1,2,3,4]. Owing to a lack of progress in oncology, the search for new treatment strategies and biomarkers has turned to the analysis and application of non-coding RNAs (ncRNAs) [5,6,7]. ncRNAs are a class of both short and long RNAs that do not code for proteins, but play a crucial role in gene regulation as well as in other cellular processes [5,6,8]. One of the newly described groups of ncRNA molecules are YRNAs, which are involved in the initiation of chromosomal DNA replication and Ro60 protein activation, by the formation of Ro60 ribonucleoprotein and binding to LA protein, which is essential for the efficient termination of RNA polymerase III [5,6,7,8]. Under-expression of YRNA1 has already been noted in prostate cancer tissue [5] and overexpression has been reported in cases of clear cell renal carcinoma [6]. YRNA1 has also been found to be dysregulated in breast cancer and in the serum of HNSCC patients [6]. The inhibition of YRNA1 and YRNA3 has been associated with decreases in cell proliferation [6] and, in bladder cancer, YRNA1 expression predicts patient survival [7]. ncRNAs are easily found and extracted from patient’s serum and plasma, making them highly promising biomarkers [5,6,7].

In our study, we found significantly decreased expression levels of YRNA1 in HNSCC cell lines when compared with a DOK cell line, which we assumed as the model of dysplastic oral mucosa cells. The lowest expression rate was found in the FaDu cell line, which is known to be the most aggressive and invasive HNSCC cell line [17]. YRNA3, YRNA4, and YRNA5 showed results similar to those of the DOK cell line, though these findings were not statistically significant. On the other hand, in the case of a comparison between patients’ tumour samples and matched healthy samples, the expression level of YRNA1 was significantly downregulated, confirming its influence on cancer progression. Similarly, in prostate and bladder cancer tissues, expression of YRNAs (YRNA1, YRNA3, YRNA4, and YRNA5) has been found to be significantly downregulated in comparison with samples from healthy tissue [5,7]. Unfortunately, there is a lack of a research concerning YRNAs in HNSCC cell lines or patient samples. Only one study, based on NGS analysis, has indicated that most YRNAs in the serum of HNSCC patients were downregulated and that only a few were upregulated in comparison with levels in healthy individuals [18].

We found that the expression levels of YRNA1, YRNA3, YRNA4, and YRNA5 were similar in various tumour localizations, and we observed the same results in the case of YRNA1 data from TCGA patients. We also analysed YRNA1, YRNA3, YRNA4, and YRNA5 expression levels according to T stage and found that, the higher the T stage, the higher the expression level of YRNAs. This phenomenon shows strong correlation between tumour growth and YRNA expression rates. In the case of other parameters such as age, G stage, N stage, M stage, and localizations, no dysregulations were found. An analysis of clinical-pathological parameters for TCGA HNSCC patients indicated significant differences in the case of HPV p16 infection status. HPV positive patients showed significantly increased YRNA1 expression levels. These results point to a correlation between YRNA1 expression rates and HPV infection, which may be caused by DNA virus integration or by other processes connected with the presence of the virus, though there are no previous studies considering the interactions of YRNA and HPV infection. Interestingly, HPV positive HNSCC patients show a better response to therapy and improved survival [19,20]. For the remainder of the analysed clinical-pathological parameters in HNSCC TCGA patients, no connections with YRNA1 were identified.

Our results have confirmed those of previous studies considering YRNAs as potential biomarkers [7,18]. Performing the ROC analysis, we found that YRNAs are highly specific in patient samples. YRNA1 is the most specific and sensitive of all examined YRNAs, demonstrating its potential use as a biomarker to distinguish between cancer and healthy tissues.

Disease-free survival and overall survival among TCGA patients were also examined. It was noted that patients from the medium + high YRNA1 expression group showed longer disease-free survival, as well as overall survival. Unfortunately, there are no studies considering DFS and OS in HNSCC. However, for a medium + high YRNA3 expression group of clear cell renal carcinoma patients, it was found that DFS and OS showed similar results, whereas YRNA4 showed opposing data [6]. It is worth mentioning that YRNA3 and YRNA4 levels showed no statistical significance. In bladder cancer, DFS and OS were found to be improved in patients with high expression values of YRNA1, YRNA3, and YRNA4 [7], while there was no difference in survival in the context of YRNA5 [7]. These outcomes show that DFS and OS vary by disease and by YRNA type. In our study, only YRNA1 was examined, because of the lack of any other data available from the TCGA database.

Little is known about the cellular functions of YRNAs in different kinds of cancer and there is no information in the case of HNSCC. Some studies have indicated that the main functions of YRNAs are in the initiation of chromosomal DNA replication [5,6,7,8], which is correlated with cell proliferation. Previous studies have also shown similar correlations, where inhibition of YRNA1 and YRNA3 resulted in decreased cell proliferation [6].

In order to explain the biological role of YRNA1 in HNSCC, and to evaluate correlated genes with YRNA1, an in-silico analysis was performed. It was observed that YRNA1 potentially targeted many genes that are involved in crucial processes for cancer development such as tumor metastasis, cancer stem cell development, apoptosis, epithelial to mesenchymal transition, and the cell cycle. For example, in the case of tumour metastasis, in the group of patients with higher expression levels of YRNA1, downregulation of the FAT1 gene was noticed. FAT1 is considered to be a tumour suppressor in HNSCC [21]. This phenomenon is correlated with higher invasiveness and migration in HNSCC cell lines [21]. Our analysis revealed that higher expression of YRNA1 is correlated with tumour growth and T stage. As FAT1 is downregulated and correlated with both invasiveness and migration, it follows that there should be a correlation in HNSCC between FAT1 and YRNA1 [21].

Finally, a gene set enrichment analysis showed that the high expression YRNA1 group was not enriched in any gene set, however, the low expression YRNA1 group was highly enriched in protein secretion processes, epidermal growth factor receptor binding, the RB-dependent pathway, the EIF4E-dependent pathway, the ERBB2-dependent pathway, the VEGF-dependent pathway, the EGFR-dependent pathway, and the cAMP-dependent pathway. These pathways are highly correlated with processes that occur during cancer development, such as enhanced proliferation, differentiation, and angiogenesis [22,23,24,25,26]. Moreover, the enhanced EIF4E-dependent pathway, which is responsible for translation initiation, was previously indicated to be overexpressed in the FaDu cell line, which is known to be the most aggressive HNSCC cell line [17]. This outcome shows that YRNA1 influences HNSCC formation and development.

To sum up, little is known about the expression of YRNAs in cancer. Only a few studies concerning this type of ncRNAs have been performed in either tissues or cancer cell lines, and there is a lack information about HNSCC. It has been noted that expression of YRNAs differs significantly between tumour and healthy tissues in many cancer types, such as bladder cancer [7], prostate cancer [5], or clear cell renal carcinoma [6]. Some studies have indicated the presence of YRNAs in serum and plasma and their utility as biomarkers.

Our study showed that YRNA1 may play an important role in the development of HNSCC and could be used as a biomarker. This is the first report wherein YRNA1 has been linked to important cellular processes such as tumor metastasis, cancer stem cell development, apoptosis, epithelial to mesenchymal transition, and the cell cycle. Moreover, the potential role of YRNA1 as a molecular sponge was considered and our results seem to confirm this. However, in this case, further studies should be performed to fully understand the role of YRNA1 in these processes and determine its usefulness as a future diagnostic molecule or even a therapeutic target.

## 5. Conclusions

The downregulated expression of YRNA1 was found in different HNSCC cell lines as well as in patients’ tumour samples. No differences were found in the expression of YRNAs at different tumor localizations, however, expression of YRNA1 was found to be significantly upregulated in stage T4 tumors. What is more, the high sensitivity and specificity of YRNA1 makes it a likely HNSCC biomarker. YRNAs are also easily isolated from serum, blood, and plasma, making it easy to obtain. TCGA data analysis also showed that YRNA1 may function as an HPV infection indicator. It was also found that higher expression of YRNA1 is associated with longer DFS and OS in HNSCC patients. Furthermore, YRNA1 expression is associated with changes in the expression of many genes involved in carcinogenesis. GSEA analysis showed enrichment in the YRNA1 low expression group in eight processes correlated with cancerogenesis. What is more, the EIF4E-dependent pathway was found to be significantly enriched in the YRNA1 low expression group, showing the strong influence of YRNA1 on HNSCC formation and development. Interestingly, high expression of EIF4E was previously indicated in FaDu cell line, which is known to be the most aggressive HNSCC cell line. This was a novel study concerning the role of YRNAs in HNSCC and their possible roles as biomarkers. YRNAs seem to play a crucial role in HNSCC development as well as in the regulation of other genes responsible for carcinogenesis. More studies must be performed to confirm their usefulness in the treatment of HNSCC.

## Figures and Tables

**Table 1:** YRNA1 expression levels in relation to clinical-pathological parameters for FFPET of head and neck squamous cell carcinoma (HNSCC) patients; T-test; Mann–Whitney test; one-way analysis of variance (ANOVA) with Dunn’s multiple comparisons post-test; *p* < 0.05 was considered to be significant. Varying patient numbers reflect availability of the data.

Parameter	Group	Mean ± SEM	*n*	*p*-Value
Age	≤70	0.03773 ± 0.009145	35	0.4873
	>70	0.06560 ± 0.0444	29	
T Stage	T1	0.04212 ± 0.01625	6	0.0211
	T2	0.03231 ± 0.01764	19	
	T3	0.03344 ± 0.01369	20	
	T4	0.1829 ± 0.1251	10	
N Stage	N0 + N1	0.0435 ± 0.01017	44	0.1747
	N2 + N3	0.1319 ± 0.1172	11	
Grade	G1 + G2	0.06342 ± 0.02687	50	0.5764
	G3 + G4	0.03474 ± 0.01272	9	
Localization	Oral Cavity	0.04271 ± 0.01368	21	0.4274
	Pharynx	0.01781 ± 0.004761	13	
	Larynx	0.1049 ± 0.05659	23	

**Table 2:** YRNA1 expression levels according to clinical-pathological parameters for all three HNSCC localizations from the TCGA data set; T-test; Mann–Whitney test; one-way ANOVA with Dunn’s multiple comparisons post-test; *p* < 0.05 considered as significant. Varying patient numbers reflect the availability of data. HPV, human papilloma virus.

Parameter	Group	Mean ± SEM	*n*	*p*-Value
Age	≤62	−1.931 ± 0.1019	65	0.7491
	>62	−2.001 ± 0.1126	51	
Sex	Female	−2.134 ± 0.1562	27	0.0932
	Male	−1.910 ± 0.0857	89	
Alcohol	Yes	−1.977 ± 0.0957	81	0.3472
	No	−1.887 ± 0.1258	32	
Smoking	Yes	−1.983 ± 0.1104	38	0.7405
	No/Ex	−1.950 ± 0.1022	75	
Cancer Stage	I + II	−1.967 ± 0.1774	15	0.8773
	III + IV	−1.973 ± 0.0864	85	
T Stage	T1	−2.064 ± 0.2855	6	0.9603
	T2	−1.963 ± 0.1214	32	
	T3	−2.030 ± 0.1783	21	
	T4	−1.947 ± 0.1265	44	
N Stage	N0 + N1	−2.129 ± 0.1038	45	0.1597
	N2 + N3	−1.896 ± 0.1124	47	
Grade	G1 + G2	−1.972 ± 0.0955	71	0.9464
	G3 + G4	−1.974 ± 0.1222	43	
Perineural Invasion	Positive	−1.969 ± 0.1331	40	0.3927
	Negative	−1.855 ± 0.1322	38	
Lymph Node Dissection	Positive	−1.800 ± 0.1763	90	0.2735
	Negative	−2.010 ± 0.0836	25	
Angiolymphatic Invasion	Positive	−1.906 ± 0.1416	32	0.3307
	Negative	−2.057 ± 0.1207	41	
Disease Surgical Margin Status	Positive	−1.879 ± 0.1469	28	0.369
	Negative	−1.990 ± 0.0947	75	
HPV p16 Status	Positive	−1.150 ± 0.2755	11	0.0002
	Negative	−2.330 ± 0.1236	16	

## Abbreviations

HNSCC	head and neck squamous cell carcinoma
TSCC	tongue squamous cell carcinoma
OSCC	oral squamous cell carcinoma
LSCC	laryngeal squamous cell carcinoma
NSCC	nasopharyngeal squamous cell carcinoma
lncRNA	long non-coding RNA
qRT-PCR	quantitative reverse transcriptase PCR
PCR	polymerase chain reaction
FFPET	Formalin-Fixed Paraffin-Embedded Tissue
TCGA	The Cancer Genome Atlas
SEM	standard error of the mean
EMT	epithelial to mesenchymal transition
HPRT1	hypoxanthine phosphoribosyltransferase 1
B2M	beta-2 microglobulin
FDR	false discovery rate
ROC	receiver operating characteristic
AUC	area under the curve
DFS	disease-free survival
OS	overall survival
RB	retinoblastoma protein
EIF4E	eukaryotic translation initiation factor 4E
ERBB2	erb-B2 receptor tyrosine kinase 2
VEGF	vascular endothelial growth factor
EGFR	epidermal growth factor receptor
cAMP	cyclic adenosine monophosphate
NGS	next generation sequencing
